# Immunity with Reference to Some of Its Relations to Surgery

**Published:** 1912-11

**Authors:** Ludvig Hektoen

**Affiliations:** Director of the Memorial Institute for Infectious Diseases; Chicago


### BUFFALO MEDICAL JOURNAL

VOL. LXVIII NOVEMBER, 1912 No. 4

### Immunity

### With Reference to Some of Its Relations to Surgery

Being the Harrington Lectures before the Alumni Association of the University of Buffalo, May 29 and 30, 1912.

By LUDVIG HEKTOEN, M. D.
Director of the Memorial Institute for Infectious Diseases
Chicago

(Concluded from last month.)

ISOAGGLUTINATION IN RELATION TO TRANSFUSION OF BLOOD.

We usually think of the antibodies of an animal as directed against cells and proteins that are quite foreign to the species to which the animal belongs. The blood of a normal animal may contain, however, antibodies for the red corpuscles of other members of the same species, and we speak of these bodies as isoagglutinins, isolysins, isopsonins. Such isoantibodies occur in human blood and on account of the significance this may have in direct transfusion, which is being practiced quite extensively, a brief review of the chief facts seems warranted. At present individuals may be separated into four distinct groups on the basis of isoagglutination.—Ottenberg.

Group 1.—The serum agglutinates the red corpuscles of the other three groups; the corpuscles, however, are not agglutinated by human serum.

Group 2.—The serum agglutinates the red corpuscles of groups 3 and 4, and the corpuscles are agglutinated by the serum of groups 1 and 3.

Group 3.—The serum agglutinates the corpuscles of groups 2 and 4, while the corpuscles are agglutinated by the serum of groups 1, 2, and 4.

Group 4.—The Serum does not agglutinate human corpuscles, but the corpuscles are agglutinated by the serum of the other groups.

The agglutinative peculiarities of each person appear to be quite permanent. It is said that in their transmission from generation to generation they follow Mendel's law. The observations at hand indicate that approximately 50 per cent. of all persons belong to the first group, 30 per cent. to the second group, 18 per cent. to the third, and 2 per cent. to the fourth. Isolysins appear to occur in about 25 per cent. of adult persons, the relative frequency being the same in health and disease, but no such grouping has been worked out for isolysis as for isoagglutination.

Now the question of practical interest is whether indiscriminate transfusion from man to man is at all in any way dangerous on account of the occurrence of isoagglutination or isolysis within the vascular system of the recipient, Crile[2] who has done such excellent work on transfusion expresses the opinion that there is practically no danger on this account; in a large number of transfusions he has seen only one instance of any indications of hemolysis. Cases are described, however, in which transfusion has been followed by untoward symptoms, and even by death, in the production of which there is good reason to believe that hemolysis and agglutination took part. Pepper and Nisbit[3] report that in a case of severe anemia from bleeding of the gums, two transfusions, each from a separate donor, were made 48 hours apart; soon after the second transfusion jaundice and hemoglobinuria developed, and the patient died in a few days. In this case there was possibility for interaction on part of the blood from three individuals. Hopkins[4] in a case of severe anemia of the pernicious type, practised transfusion with a blood, the corpuscles of which were agglutinated by the serum of the patient; immediately after the transfusion, which lasted half an hour, the patient became irrational and comatose with hemiplegia, and died after nine hours. In this case phagocytosis of red corpuscles by leukocytes was observed in the blood, and after death the serum of the patient did not agglutinate the corpuscles of the donor, indicating that the agglutinins had been absorbed by the transfused corpuscles. Is it not highly probable that death in this instance resulted from intravascular agglutination? After intravenous injection of 50cc. of blood, the corpuscles of which were agglutinated by the serum of the patient, Schultz[5] saw collapse with loss of consciousness, vomiting and diarrhea, fever and edema of the hands and feet. Ottenberg[6] mentions that in two transfusions of blood, the corpuscles of which were agglutinated by the serum of the recipient, there was phagocytosis of the red corpuscles in the circulation; in one of these instances the agglutinin of the patient's serum disappeared probably from

2 Hemorrhage and Transfusion, 1909.

A Case of Fatal Hemolysis following direct Transfusion of Blood by Arteriovenous Anastomosis. Contrib. from the Wm. Pepper Laboratory of Clinical Medicine, 1907-8, 6.

4 Arch. Int. Med., 1910, 6, p. 270.

5 Ueber Bluttransfusion beim Meuschen unter Berucksichtigung biologische corprüfungen. Berl, Kl. Wchnsehr., 1910, 47, p1407.

6 Transfusion and the Question of Intravascular Agglutination, Jour. Exper. Med., 1911. 13, p. 425.

Also private communication.

absorption by the transfused corpuscles. Both the patients died but whether from effects of the transfusion is uncertain.

From the facts at hand it seems to me that the possibility of isohemolysis and of isoagglutination must be taken account of in transfusion. It is true that homologous transfusions rarely have been followed by untoward symptoms even though the blood concerned in many cases must have been agglutinative and lytic; this no doubt depends in part on there being perhaps too little agglutinin and on the excess of agglutinable cells as would be the case especially when the donor's serum is agglutinative for the patient's corpuscles. The most dangerous combination is illustrated by the cases recited, namely agglutination of the donor's corpuscles by the patient's serum; now if isolysis were added the dangers would be increased. As no surgeon would wish to take any chances in matters of this nature, the donor in transfusion better be selected by appropriate tests. The donor should be of the same agglutinative group as the patient; there should be no isolysins in the bloods. In some hospitals tests of this kind[7] are made for all transfusions. Corpuscles agglutinated by the recipient should be avoided also because they appear to be readily taken up by the leukocytes and removed from service. As Richard Lower expressed it in 1665, transfusion should be practised in order if possible that those who "want blood or have corrupt blood may be supplied from others with sufficient quantities and such as is good." In urgent cases when the donor cannot be selected by proper tests, the possible dangers of transfusion must be balanced against the dangers of the condition without any transfusion.

### Allergy.

From all that has been said we naturally would expect that the protective reactions induced by an antigenic substance would be stimulated and reenforced on the reintroduction of the antigen, but it would seem, at any rate at first glance, as if under certain circumstances this is not the case because peculiar, often violent, and even fatal symptoms may set in when the antigen in question is introduced again. This phase of immunization has been studied very actively of late under the headings of anaphylaxis (Richet), which means the reverse of prophylaxis, of hypersusceptibility, and of allergy (v. Pirquet) which means changed reactivity and which in my opinion is the most desirable designa-

---

7 The tests may be carried out by mixing say .1 cc. of serum with .1 cc. of a 5 per cent. suspension of corpuscles and adding salt solution sufficient to make .5 or 1.0 cc. in all. The tubes are incubated at 37d. C. for two hours. At this time it can readily be seen whether any laking has occurred; agglutination when present is recognized by the clumping and irregular sedimentation of the corpuscles. The serum of the prospective donor must be tested with corpuscles of the recipient and the serum of the recipient with the corpuscles of the donor.

tion. On account of the importance which allergy now occupies in diagnosis and in our conceptions of the intimate processes of infectious and other diseases, I have deemed it well to review briefly some of the chief facts in regard to its nature and its manifestations.[8]

### The Experimental Basis.

It would not be feasible to go into details in regard to the vast amount of work on the results of which rests our present understanding of allergy. As a rule an animal, once thoroughly subjected to the influence of a foreign protein, be it of vegetable, animal, or bacterial origin, but which must be introduced as such into its blood or tissues, develops a condition which manifests itself by special symptoms when the protein is reintroduced. It takes some time, at the very least a few days, for this condition to develop; gradually a maximum intensity is reached after which there is a slow return to the normal, a curve being traced that is like the antibody curve. Allergy may be established with respect to many protein substances, notably serum, bacteria and their derivatives, milk, egg-white, red corpuscles and various more or less pure proteins. These are all substances with antigenic powers and the allergic reactions follow the same laws as to specificity as antibody-antigen reactions in general. A passive allergy, analogous to passive immunization, is obtainable by the injection of a normal animal, particularly the guinea-pig, with the serum of animals or persons already allergic or sensitized, (Gay and Southard, Otto) and this transferred condition is specific in the same way as the allergy of the donor. This demonstration, which clearly shows that allergy depends on specific substances in the blood, is the basis of the conception of the nature of allergy which now prevails, namely that on the introduction of the antigen new substances, antibodies of the general structure of amboceptors, are developed, which unite the complement with the antigen, the complement by virtue of its digestive powers now setting free substances that in proper concentration can cause the symptoms of allergy. In other words, the action is regarded as one of specific proteolysis in the blood and tissues, the toxic substances thus produced being split-products or possibly preformed poisons (endotoxins) that are set free. Substances causing the general symptoms of anaphylactic or allergic intoxication in guinea-pigs are obtainable in vitro by splitting up bacteria and egg-white with chemicals (Vaughan); by the acton of allergic or immune serum and complement on

8 For a more detailed discussion of allergy, see my paper in Jour. Amer. Med. Assn., 1912, 53, p. 1081.

the corresponding antigen (Friedemann, Friedberger); by the action of complement on the precipitate produced in serum by specific precipitin (Friedberger); and by letting bacteria like the pneumococcus, the gonococcus, and the like, digest themselves (Rosenow). At present then, our explanation of the phenomena of allergy centers about the view that parenteral protein digestion plays the essential part in their production, the digestion being accomplished by an amboceptor-complement mechanism.

The symptoms of the allergic reaction may vary a great deal depending on the species and also on the quantity of toxic substances. In the guinea-pig the reinjection of the antigen may give rise to explosive symptoms, death resulting in a few moments from asphyxia due to spasm of the bronchi; when death does not occur immediately there is a marked fall of the temperature, and when the reaction is very mild there may be fever. In dogs a noteworthy result is marked fall of blood pressure due to splanchnic action, and in rabbits occur respiratory disturbances, prostration, and a cardiac rigor; sometimes local changes of edema and necrosis develop. Common in all these conditions is temporary loss of complement, loss of coaguability of the blood and leukopenia.

### Allergic Phenomena in Man.

A variety of allergic phenomena occur in a man. In the first place the injection of therapeutic serum in highly exceptional instances may cause severe dyspneis symptoms not unlike those observed in the acute reaction in guinea-pigs; just how sensitization has occurred in such instances is still a matter of speculation. More familiar is the reaction called "serum-disease" which sometimes appears from 8-12 days after the injection; in this case we assume that part of the serum remains unchanged until enough antibody has developed to cause a sharp reaction. If serum is reinjected after some time, thus reproducing the experimental conditions, the reaction may develop almost immediately or after three or four days. The symptoms are familiar; urticaria, edema, articular pain, swelling of the lymph nodes, leukopenia, fever, weakness, and sometimes collapse. In the second place, human diseases present a rich field for the study of allergic phenomena to some of which attention may be directed for a few moments. In infectious diseases, especially those which do not, like diphtheria and tetanus, depend practically altogether on the secretion and absorption of typical toxins, there is much to indicate that many of the symptoms result from the interaction of the antibodies of the host and the microbes, that the tymptoms are, so to speak, allergic manifestations due to toxic substances arising or set free in the course of the destruction of the microbes. In the light of this view the incubation period would represent the time necessary for the formation of sufficient antibodies to break up the microbic proteins. In many infections this period corresponds closely in length to the period preceding the acme of antibody formation under experimental conditions. This is illustrated especially well in small-pox and vaccinia, and as here the cutaneous lesions contain the virus it is reasonable to regard the lesions as the result of the reactions between the antibodies and the virus. On daily vaccinations of a susceptible person, v. Pirquet found that the miximum reactions occur at the same time about all points of inoculation, namely when it occurs at the first point; here, then, the antibodies educed by the first vaccination on reaching the required concentraction act in the same way and with the same result on the virus deposited in the skin from day to day. We have just heard that in serum disease the incubation may be shortened very greatly in persons previously injected with serum, that is sensitized or partially immunized, and so also on revaccination of previously vaccinated persons, accelerated reactions often occur; and varioloid is to be regarded as an atypical form of small-pox in a partially immune person. Undoubtedly the incubation and clinical course of other infections is influenced by heightened reactivity on the part of the body caused by previous attacks.

Jenner probably was the first to recognize what we now speak of as allergic reactions to infectious material. He observed that the skin of persons having had small-pox or cow-pox reacted peculiarly to the application of variolous matter Thus in a woman who had cow-pox 31 years before she was inoculated with variolous matter, "an efflorescence of palish, red color soon appeared about the parts where the matter was inserted and spread itself rather extensively, but died away in a few days without producing any variolous symptoms." That common wound infection is excluded seems clear because he observed the phenomenon so regularly under these special conditions that he was led to comment as follows: "It is remarkable that variolous matter, when the system is disposed to reject it, should excite inflammation on the part to which it is applied more speedily than when it produces the small-pox. Indeed it becomes almost a criterion by which we can determine whether the infection will be received or not. It seems as if a change, which endures through life, had been produced in the action, or disposition to action, in the vessels of the skin; and it is remarkable too, that whether this change has been effected by the small-pox, or the cow-pox, that the disposition to sudden cuticular inflammation is the same on the application of variolous matter." In this remarkably clear statement we note with special interest the intimation of the diagnostic value of the reaction.

The specific allergic state of the infected body is shown then also by the reactions which take place when the substance causing the infection, the antigenic substance, is introduced into it. The tuberculin test and other tests like it are based on this circumstance. Reaction to tuberculin is developed best perhaps in the tuberculous person or animal, but it is obtainable also after active sensitization by means of tubercle proteins, and passive transfer of the reaction to guinea pigs has been accomplished in such a variety of ways that it unquestionably must be regarded as humoral in nature. Of the different forms of the tuberculin reaction it is not necessary to speak. Since the introduction of vaccine therapy, general reactions with fever and malaise, acute changes in local lesions, and sometimes reactions at the site of the injection of the vaccine have been observed in many subacute and chronic infections; diminution of complement and a fall in opsonin—negative phase—generally accompany this reaction. Within the past few months, Noguchi has shown that in syphilitics, extracts of the treponema pallidum, which he has grown in pure culture, give a characteristic cutaneous reaction. Highly interesting observations have been made recently on the allergic reactions in gonococcal infections. We now know that the serum of patients with such infections contains substances that in conjunction with gonococci bind complement. When inoculation of gonococci was begun it was soon observed that acute reactions may develop in the lesions of inoculated patients. Irons (1908) described in detail the reaction as it appears after the injection of gonococci in arthritis. This reaction which in every way is analogous to the general tuberculin reaction is characterized by "a rise in temperature, often only slight, an increase in pain and tenderness in the affected joints, and occasionally some increase in swelling, and a variable degree of malaise. The symptoms follow the injection in from 8 to 12 hours and commonly last 24 hours. Frequently there is a decided tenderness at the site of the injection, greater than after the inoculation of the same dose of the same proportion in normal subjects. Commonly there is marked redness and edema lasting from 24 to 48 hours." Uusually there is a slight leukocytosis during the first twenty-four hours. A similar general reaction with increased pain and swelling of the focal lesions occurs in other forms of gonococcal localizations. Quite recently, Irons has demonstrated that in gonococcal infections a cutaneous reaction is easily obtainable by applying extracts of gonococci to the skin in the same way as tuberculin is applied in v. Pirquet's test.[9]

It is self evident that as the details of these newer allergic reactions are worked out, useful adjuvants will be added to our means of diagnosis of occult infections.

That many so-called idiosyncrasies with respect to certain foods,—strawberries, cheese, fish, oysters, buckwheat, pork, eggs, cow's milk, etc.—in reality are expressions of allergy can hardly be doubted. Some striking examples of sensitiveness to milk and egg-white ("egg asthma") have been described. It is true that not many cases of alimentary allergy, or anaphylaxis have been studied thoroughly from this newer point of view as to their nature, but in one case of intolerance of pork it was found that the serum of the patient introduced in guinea-pigs produced allergy to pork (passive anaphylaxis).

It has been suggested that under certain conditions the proteins of the body itself may become so changed—"heterologized"—as to acquire antigenic powers and on reacting with the antibodies give rise to toxic substances. It would be particularly at the seat of severe lesions, such as are produced in certain forms of chronic tuberculosis, and in burns, for instance, that this change most likely would occur. Claiming support from experiments, Heyde[10] would account in this way for the fall of temperature, convulsions and delirium that sometimes develop unexpectedly several days after a burn. Certain drug eruptions, e. g. those from iodid and bromid, may rest on a similar basis. Since it has been claimed that placenta, amniotic fluid, and fetal serum act as foreign proteins in adults of the same species, the idea that puerperal eclampsia, "the disease of theories," is an anaphylactic condition has gained much ground.

The skin, as we have seen, presents many interesting phenomena from the allergic point of view: the lesions of serum disease, the eruptions of many infectious diseases, the urticarias and other manifestations of the absorption of foreign proteins from the digestive tract (alimentary anaphylaxis), the allergic reactions of infectious diseases, all indicate that the skin not only has a marked affinity for foreign proteins but that it also is a

9 The serum of animals immunized against gonococci has been found to harbor various specific antibodies—agglutinins, precipitins, bactericidal and opsonic elements, and bodies that in connection with gonococci bind complement—and from the study of the action of such serum, on various strains of gonococci it appears that the gonococcus group is made up of races with distinct affinities, so that the extracts or mixtures of gonococci used for diagnostic purposes better be derived from several distinct strains. It is interesting to note also that there is a chemical relationship between the gonococcus and the meningococcus because it does not seem possible to differentiate absolutely one from the other by means of the complement fixation test; this also seems to hold true with respect to the cutaneous reaction.

10 Med. Klink, 1912. 8, p. 263.

sensitive reagent for the various products of parenteral proteolysis. To what extent other tissues have similar relations cannot be discussed now, but I would mention that hay fever and certain forms of asthma appear to be allergic phenomena, and that certain hemorrhagic lesions of the intestines also may be of this nature.

ALLERGY AND IMMUNITY.

Returning now to the apparent incompatibility between immunity and the allergic state, we observe that in the so-called sensitized body the specific foreign protein may be split up so rapidly by the complement, acting through the specific amboceptor, that toxic substances are set loose in toxic quantities; in the normal body symptoms develop probably because the process proceeds slowly and gradually. Now, infectious microbes are destroyed by the same mechanism of proteolysis, and when small quantities are introduced, as often must be the case in natural infections, the microbes may be disposed of in this way before they multiply sufficiently to supply toxic doses of poison, and the process of destruction will occur with greater promptness in the sensitized, that is more or less immunized, body "when the system is disposed to reject" the foreign protein. This is immunity, natural and acquired, based largely on prompt, early reaction. In reality the allergic state is not one of greater susceptibility as we frequently hear it stated, but one of greater reactive, and consequently in infectious diseases of greater protective power, the actual susceptibility to the same poisons being probably the same in the average allergic and normal body. Hence there is no incompatibility between immunity and allergy; the allergic symptoms are manifestations of antibody-antigen reactions in the living body.

